# Measuring the clustering effect of the SARS-CoV-2 transmission in a school population: a cross-sectional study in a high incidence region

**DOI:** 10.1038/s41598-023-42470-x

**Published:** 2023-09-28

**Authors:** Milton Severo, Paula Meireles, Ana Isabel Ribeiro, Vítor Morais, Henrique Barros

**Affiliations:** 1 EPIUnit–Instituto de Saúde Pública, Universidade do Porto, Rua das Taipas, nº 135, 4050-600 Porto, Portugal; 2 Laboratório Para a Investigação Integrativa e Translacional em Saúde Populacional (ITR), Rua das Taipas 135, 4050-600 Porto, Portugal; 3 Faculdade de Medicina, Universidade do Porto, Alameda Prof. Hernâni Monteiro, 4200-319 Porto, Portugal; 4 Instituto de Ciências Biomédicas- Abel Salazar, Universidade do Porto, Rua de Jorge Viterbo Ferreira, 228, 4050-313 Porto, Portugal

**Keywords:** Diseases, Health care, Risk factors

## Abstract

Since the beginning of the pandemic, there has been a great deal of controversy regarding the role of schools in the spread of SARS-CoV-2 infection, and the relative contribution of students, teachers, and others. To quantify the clustering effect of SARS-CoV-2 infection within classes and schools considering the seroprevalence of specific antibodies among students and school staff (teachers and non-teachers) evaluated in schools located in the Northern region of Portugal. 1517 individuals (1307 students and 210 school staff) from 4 public and 2 private schools, comprising daycare to secondary levels, were evaluated. A rapid point-of-care test for SARS-CoV-2 specific IgM and IgG antibodies was performed and a questionnaire was completed providing sociodemographic and clinical information. We calculated the seroprevalence of IgM and IgG antibodies and estimated the Median Odds Ratio (OR) and 95% confidence interval (CI) to assess the clustering effect, using a multilevel (school and class) logistic regression. SARS-CoV-2 seroprevalence (IgM or IgG) was 21.8% and 23.8% (*p* = 0.575) in students and school staff, respectively. A total of 84 (8.6%) students and 35 (16.7%) school staff reported a previous molecular diagnosis. Among students, those who reported high-risk contacts only at school (OR = 1.13; 95% CI 0.72–1.78) had a seroprevalence similar to those without high-risk contacts; however, seroprevalence was significantly higher among those who only reported a high-risk contact outside the school (OR = 6.56; 95% CI 3.68–11.72), or in both places (OR = 7.83; 95% CI 5.14–11.93). Similar associations were found for school staff. The median OR was 1.00 (95% CI 1.00, 1.38) at the school-level and 1.78 (95% CI 1.40, 2.06) at the class-level. SARS-CoV-2 seroprevalence was similar between students and staff, without a clustering effect observed at the school level, and only a moderate clustering effect documented within classes. These results indicate that the mitigation measures in the school environment can prevent the spread of class outbreaks to the remaining school community.

## Introduction

Since the identification of the severe acute respiratory syndrome coronavirus 2 (SARS-CoV-2) in December 2019^1^, most countries implemented measures of school closure. This involved nationwide or regional closures and total or partial closure of schools (in which schools were either closed for some grade levels or were working with restricted capacity). In March 2020, the United Nations Educational, Scientific and Cultural Organization estimated that more than 100 countries had implemented national school closures^2^, and in April 2020, more than 90% of the students worldwide were in remote learning^3^.

The rationale for school closures followed evidence from the influenza outbreaks where the reduction of social contact between students led to a reduction of the transmissibility index^4^. It was assumed that this holds for SARS-CoV-2. However, school closures can be accompanied by increased social mixing of children and adults and among children from different schools^5^, which might undermine the expected effects. In addition, school closures—particularly if prolonged—widen inequalities, disproportionately affecting socially disadvantaged children and adolescents. These groups are less likely to have access to digital technologies required for remote learning, to healthy and nutritious food choices, and are more likely to be exposed to social (e.g. violence) and environmental hazards (e.g. overcrowding) in the residential environment^2, 6^. So, given the possible deleterious effects of school closures, we must understand the extent of transmission in the school environment.

In fact, in the case of SARS-CoV-2 infection, the role of schools in the spreading of the infection remains controversial^7^, with some studies indicating substantial increases in case rates associated with school openings^8^, while others document a small impact^9^. Additionally, a nationwide, population-based seroepidemiological study conducted between February and March 2021 in Portugal showed that seroprevalence in children and adolescents was similar to that of persons aged between 20 and 69 years, suggesting a similar risk of infection^10^. In addition, several tracing studies within education settings identified only a few outbreaks^11, 12^.

Currently, available data did not quantify the magnitude of the effect of children-to-children transmission within classrooms and schools (the so-called cluster effects). The detection of clustering at the school or classroom would indicate that the school setting is important in understanding individual differences in infection. Therefore, this knowledge may help public health authorities to direct their efforts toward specific classrooms and schools. If no clustering is identified, this suggests that mitigation measures should be targeted at individuals, families, and social networks outside the school community.

In this study, we aimed to quantify the seroprevalence of specific antibodies to SARS-CoV-2 among students and school staff in six schools located in the Northern region of Portugal and to quantify the clustering effect of SARS-CoV-2 within classes and schools.

## Methods

### Setting and participants

The study protocol was approved by the ethics committee of the Institute of Public Health of the University of Porto (ID 20154). Participation was voluntary and written informed consent was given by the participants, or the respective legal guardian in case of those aged less than 16. All methods were carried out in accordance with relevant guidelines and regulations.

**Figure 1:**
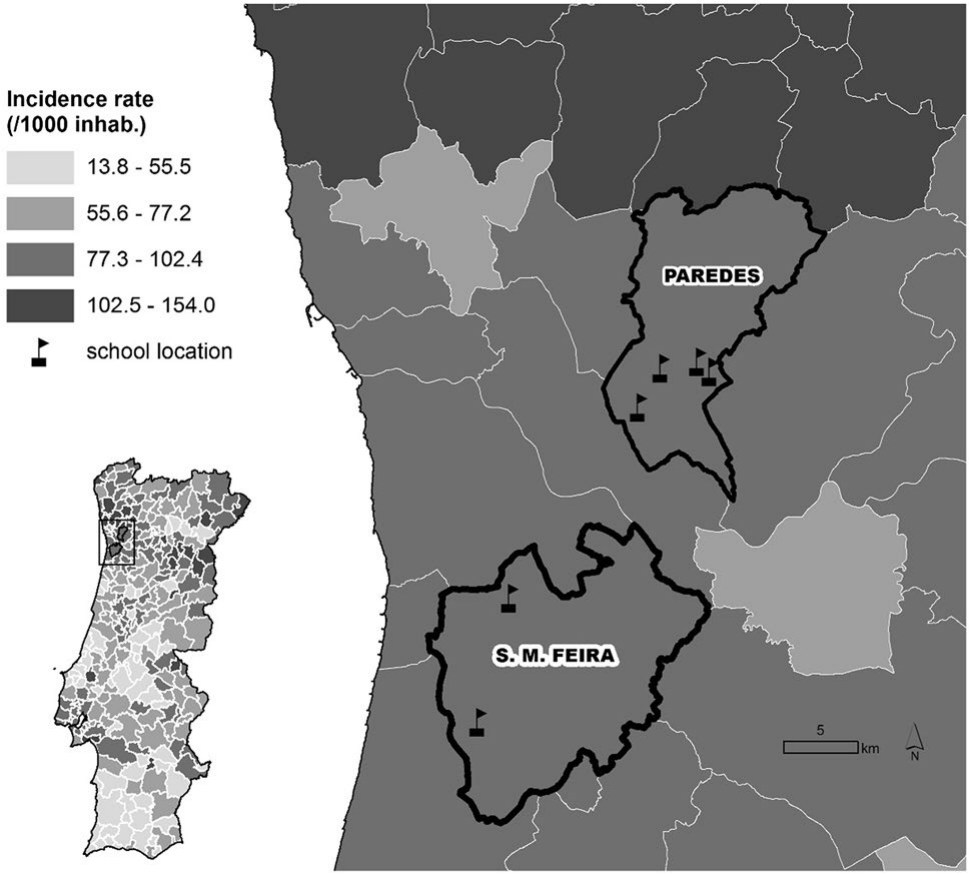
Location of the six included schools and incidence rates in the surrounding municipalities and in the municipalities where they are located from March 2020 to April 8, 2021.

In the study conducted from March to April 2021, we invited a total of six schools (four public schools and two private schools) located in two municipalities of the Porto Metropolitan Area (Santa Maria da Feira and Paredes) to participate, using quota sampling. These two municipalities were selected because they both belong to the Porto Metropolitan Area—the second largest metropolitan area of the country and one of the Portuguese regions most affected by the epidemic—and presented high incidence rates of SARS-CoV-2 as depicted in the map from Fig. 1.

All students and school staff (teachers and non-teachers) of 84 classes from the six schools were offered a serological point-of-care test for SARS-CoV-2 specific IgM and IgG antibodies between March 25 and April 9, 2021 (Fig. 1). The study was conducted prior to the vaccination of both students and school staff.

From a total of 1617 students and 253 school staff, 1307 (81%) and 210 (83%), respectively, accepted to participate and had valid tests. The students’ age ranged from 0 to 18 years old and the ages of the school staff ranged from 22 to 65 years old. Participants’ characteristics are presented in Table 1.

**Table 1:** Description of the participants.

	Students	School staff		Overall
	(N = 1307)	(N = 210)	*p*-value	(N = 1517)
Age (years)				
Mean (SD) [Min, Max]	11 (4.0) [0, 18]	47 (9.8) [22, 65]	< 0.001	18 (15) [0, 65]
Missing	370 (28.3%)	3 (1.4%)		373 (24.6%)

Sex	N (%)	N (%)	N (%)	N (%)
Female	628 (48.3%)	179 (85.2%)	< 0.001	807 (53.5%)
Male	671 (51.7%)	31 (14.8%)		702 (46.5%)
High-risk contact				
None	606 (62.9%)	130 (64.0%)	0.060	736 (63.1%)
Inside the school only	180 (18.7%)	43 (21.2%)		223 (19.1%)
Outside school only	54 (5.6%)	9 (4.4%)		63 (5.4%)
Both	124 (12.9%)	21 (10.3%)		145 (12.4%)
Previous RT-PCR tests and diagnosis				
Never tested	512 (52.4%)	60 (28.6%)	< 0.001	572 (48.2%)
Tested, not diagnosed	381 (39.0%)	115 (54.8%)		496 (41.8%)
Tested, diagnosed	84 (8.6%)	35 (16.7%)		119 (10.0%)
IgM				
Non-reactive	1221 (93.4%)	179 (85.2%)	< 0.001	1400 (92.3%)
Reactive	86 (6.6%)	31 (14.8%)		117 (7.7%)
IgG				
Non-reactive	1032 (79.0%)	172 (81.9%)	0.330	1204 (79.4%)
Reactive	275 (21.0%)	38 (18.1%)		313 (20.6%)
IgM or IgG				
Non-reactive	1022 (78.2%)	160 (76.2%)	0.520	1182 (77.9%)
Reactive	285 (21.8%)	50 (23.8%)		335 (22.1%)

### Questionnaire

A questionnaire was completed as part of the testing procedure. Each participant was given an ID, and on the day of the testing or the previous day, the participants, if 12 or older, or their legal guardian, were invited by email to complete an online questionnaire addressing clinical aspects of SARS-CoV-2 infection and sociodemographic characteristics. The questionnaire included information on SARS-CoV-2 cases among cohabitants (parents, grandparents, children, siblings, others), and non-cohabitants and type of relationship (namely, family or friend, a colleague at school, student, teacher, other school workers); having been quarantined since beginning 2020; symptoms since the beginning of 2020 (abdominal pain, anosmia, arthralgia, asthenia, chest pain, cough, diarrhea, dysgeusia, fever, headache, myalgia, nausea, rhinorrhea, shortness of breath, sore throat); previous SARS-CoV-2 testing and diagnosis; and COVID-19 vaccination. The sociodemographic section inquired about sex, age, educational level, professional category (in the case of school staff), and number of cohabitants aged less than 10 years, between 10 and 19 years, 20 and 69 years, and aged 70 years or more.

The questionnaire used in this study was adapted from a previous version applied to university students and workers^13, 14^. To ensure its appropriateness for assessing SAR-CoV-2 symptoms, experts in infectious diseases, such as medical professionals and researchers, evaluated its content validity and face validity.

The classes were re-coded into: Daycare [1–2 years old], Preschool [3–6 years old], first cycle [1–4 years school], second cycle [5–6 years school], third cycle [7–9] and secondary [10–12 years school].

The information about known high-risk contact with SARS-CoV-2 cases was categorized into the following four categories: no contact, contact inside school only, outside school only, and at both settings.

### Seroprevalence of SARS-CoV-2 specific antibodies

To determine the presence of SARS-CoV-2 specific IgM and IgG antibodies the STANDARD Q COVID-19 IgM/IgG Combo (reported sensitivity of 94.5% based on samples obtained 7 or more days after symptoms onset and specificity of 95.7% for both IgG and IgM) was performed according to manufacturer instructions by trained researchers.

### Statistical analysis

The seroprevalence was computed as the proportion of individuals with a reactive result in the IgM or IgG band. We compared proportions using the Pearson Chi-Square or the Fisher exact test when the assumptions for the Chi-Square test did not hold. P-values lower than 0.05 were considered statistically significant.

The map from Fig. 1 was generated using the software ArcGIS Pro version 2.3.0. ArcGIS Pro is a Geographic Information System (GIS) platform developed by Esri, used for creating, analyzing, and visualizing geospatial data (For more information about ArcGIS Pro 2.3.0, you can visit the following link: https://www.esri.com/en-us/arcgis/about-arcgis/overview).

The adjusted odds ratios (aOR) were used to estimate the association between having a reactive IgM or IgG test result and known high-risk contact with confirmed cases, after accounting for sex, age, and presence of symptoms (any of the listed). The aOR and respective 95% confidence intervals were estimated using unconditional logistic regression.

Multilevel mixed-effects logistic regression was fitted with a random effect at the class- and school-level. Multilevel mixed-effects logistic regression is a statistical model that is specifically designed to analyze binary data with a nested structure. In this framework, the data is organized in a way that students are nested within higher-level units (e.g., classes and schools). This type of regression model allows for the examination of both within-group (individual) and between-group (class and school) effects. The inclusion of random effects at the class and school levels in the regression model allows us to account for the potential clustering of observations within the same class or school. In other words, students within the same class or school may be more alike than students in other classes or schools. The random effects allow for the estimation of the variability or heterogeneity between classes and schools.

Crude and adjusted median odds ratio (MOR) and the corresponding bootstrap 95% CI were calculated to quantify the magnitude of the clustering effect at the class- and school-level. The clustering effect, and the MOR, quantify the variation between clusters (second-level variation—classes, third-level—schools) by comparing two individuals from two randomly chosen clusters. When the MOR is equal to one, it means there are no differences between classrooms or between schools. The MOR can be conceptualized as the increased risk that (in median) one would have if moving to another class/school with a higher risk^15^. Multilevel models were adjusted for age, sex, presence of symptoms (any of the asked symptoms), and known high-risk contact with confirmed cases.

### Data sharing

Data and statistical analyses code can be obtained upon request from other researchers. Requests should be sent to the first author Milton Severo (milton@ispup.up.pt).

## Results

Among the 1517 participants, 86 (6.6%) out of the 1307 students and 31 (14.8%) out of the 210 school staff members were reactive for IgM. For IgG, 275 (21.0%) students and 38 (18.1%) school staff members were reactive, and 285 (21.8%) students and 50 school staff members (23.8%) were reactive for either IgM or IgG (Table 1). Previous diagnosis of SARS-CoV-2 infection was higher among school staff (16.7%) compared to students (8.6%). On the same note, over half of the students had never been tested for SARS-CoV-2 compared to 28.6% among school staff (Table 1).

**Figure 2:**
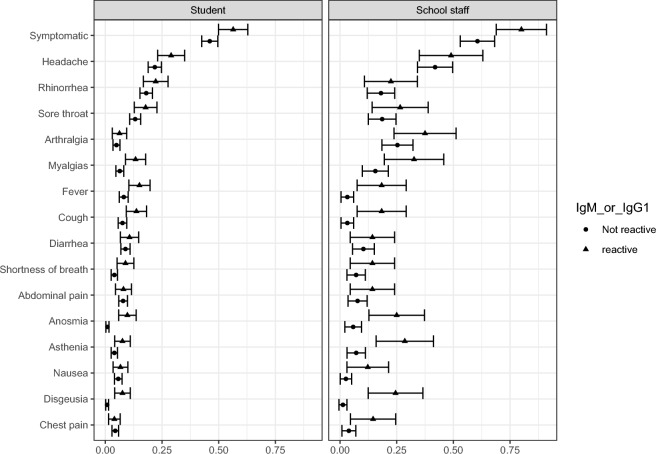
Prevalence of symptoms (CI95%) according to serological status in students and school staff.

In general, students had a prevalence of any symptoms since the beginning of 2020 of 48.4%, while in school staff it was 65.2%. Figure 2 presents the prevalence of different symptoms since January 2020, according to the results for IgM and IgG. The presence of symptoms was higher among students with a reactive result (56.4%) compared to those with a non-reactive status (46.1%). This difference was larger among school staff (80.0% compared to 60.6%). Similar results were found for the presence of dysgeusia, anosmia, myalgia, fever, and cough.

**Figure 3:**
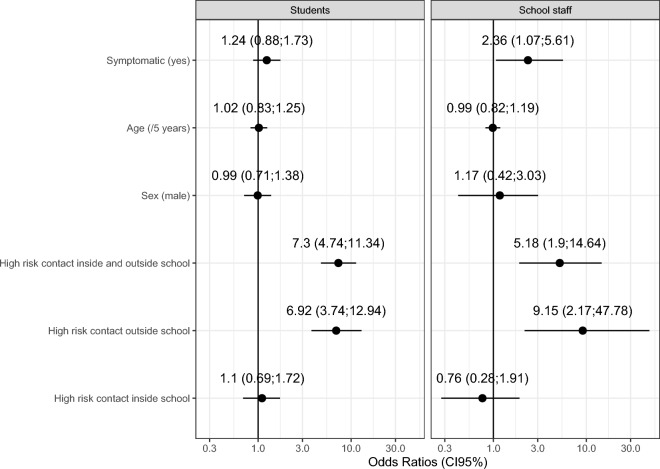
Determinants of positive antibody test (IgM or IgG) among students and school staff.

Figure 3 presents the adjusted association between having had high-risk contacts and IgM or IgG reactive status for students and school staff separately. Having had high-risk contacts inside the school only was not significantly associated with having a reactive IgM or IgG test in students (OR = 1.10; 95% CI 0.70, 1.74) and school staff (OR = 0.76; 95% CI 0.29, 1.98), while having high-risk contacts outside school only was strongly associated with having a reactive IgM or IgG test in students (OR = 6.91; 95% CI 3.73, 12.82) and school staff (OR = 9.15; 95% CI 2.04, 40.93). A similarly strong positive association was found for having high-risk contacts both inside and outside schools for both groups (students: OR = 7.30; 95% CI 4.74, 11.34; school staff: OR = 5.18; 95% CI 1.9, 14.64). There was no association with sex or age in both strata. Reporting symptoms was positively associated with being reactive (OR = 2.36; 95% CI 1.07, 5.61) among school staff, while among students no association was observed (OR = 1.24; 95% CI 0.88, 1.73).

**Figure 4:**
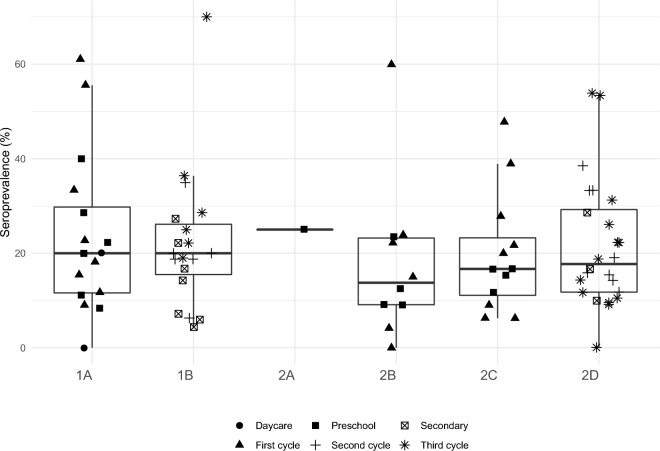
Seroprevalence of SARS-CoV-2 in students of the classes according to school. 1A, 1B: schools of region 1 (S.M. Feira); 2A, 2B, 2C and 2D: schools of region 2 (Paredes).

Figure 4 shows the seroprevalence in students within each class and school from the two municipalities. The median prevalence and interquartile range was very similar between schools, and one to two classes showed extreme values of prevalence in each school (values higher than 40% of seroprevalence within classes). From a total of 84 classes, eight (9.5%) had more than 40% of students with reactive results. The median odds ratio at the school-level was estimated at 1.00 (95% CI 1.00, 1.39) and 1.78 (95% CI 1.40, 2.06) at class-level. After adjusting for sex, age, being symptomatic, and high-risk contact (outside, inside school, or both) the median odds ratio at the school-level was estimated at 1.00 (95% CI 1.00, 1.92) and 2.20 (95% CI 1.66, 2.66) at the class-level.

## Discussion

Our study is the first that quantified the seroprevalence of specific antibodies to SARS-CoV-2 among students and school staff to assess the magnitude of the clustering effect of cases within schools and classes. The risk of infection was estimated to be 2 times higher within some classes compared to others, showing a clustering effect at class-level. This result was independent of sex, age, presence of symptoms, and high-risk contact (outside, inside school, or both), and schools. A similar study in school children in Switzerland^16^ at an earlier phase of the pandemic (July 2020) showed no significant clustering effect within classes. In this study, most classes had only a single seropositive child among the tested children. The main reason for this difference may be that the prevalence in Switzerland was very low in that phase of the pandemic (2.8%). In our study, approximately 10% of classes had more than 40% of students with reactive results, with as much as 14 reactive in 20 students in the class.

By April 2021, one-fifth of school students and staff had evidence of prior infection based on antibody seropositivity. These estimates were close to those reported in Northern France^17^, but lower than those found in England^18^, with approximately one-third of students and staff, or in the Czech Republic^19^, where almost half of the secondary students had detectable antibodies on a similar timeframe. On the other hand, our estimates were higher than those reported in Montreal, Canada^20^ (circa 6%) and Indiana, USA^21^(circa 3%). However, these studies were conducted at different time periods than ours, which limits our capacity to establish direct comparisons. Additionally, the SARS-CoV-2 epidemic waves occurred at different times in each country; therefore, the seropositivity of the population could differ even if the studies were conducted at the same time.

Our results suggest that, under circumstances of careful control of contacts and hygiene measures, the school setting play a small role in SARS-CoV-2 transmission as evidenced by the absence of a clustering effect at the school-level. The schools in our study implemented specific control measures for SARS-CoV-2, such as not mixing students from different classes. This approach could potentially explain why we did not observe significant school-level effects.

Supporting the same idea, a study conducted in 2021 in the UK, found that SARS-CoV-2 infections and outbreaks were uncommon in educational settings and that COVID-19 school incidence mirrored the incidence observed at the regional and community-level^22^.

Also, the fact that seroprevalence was 7 to 9 times higher (students and school staff, respectively) when the high-risk contact occurred outside the school only (versus 1.1 times higher when the contact occurred inside the school only) supports the idea that educational settings play a small role in SARS-CoV-2 dissemination. Even when individuals had both types of contact, the odds ratio was like that observed amongst those with high-risk contacts outside school only. Our result is similar to the one found in Milan, where no differences were found in the seroconversion rate between students who attended school compared to those who started remote learning, with 61% of students diagnosed with the infection reporting that the contact occurred within the household^23^.

Seroprevalence was similar between students and school staff, and the ratio between seroprevalence and the diagnosed cases was between 1.5 and 2.0, respectively for school staff and students. Even compared to the age-specific incidence in the municipalities (ranged from 7.2 to 11.1%) where schools were located as reported by the National Surveillance System, this ratio was between 2 and 3, which was lower than what was found in a previous study in three Portuguese public higher education schools^13^, and in the first national serological survey (ISNCOVID-19) where the ratio was approximately 5–6 times higher^24^. A possible explanation is that there was an increase in the availability of diagnostic tests and improved screening compared to the initial phase of the pandemic. This could account for the lower ratio between seroprevalence and diagnosed cases.

While we were able to evaluate over 80 classes, the inclusion of only 6 schools could have limited the capacity for detection of a school clustering effect. Nevertheless, our criteria to select the municipalities—having high incidence and belonging to Porto Metropolitan Area—increased the power of the study to detect clustering effects. Besides, the high participation rate in the study, higher than 80%, and the fact that the sample was assembled before the vaccination of school staff and students increases the relevance of the results and their strength. Finally, we did not explore the factors—e.g., class size, socioeconomic characteristics of the participants, room conditions such as ventilation, mask use^25^, sun exposure, physical distancing^26^, and hygiene—behind the clustering effect observed at the class level, which could give important insights on the additional measures that need to be implemented to prevent outbreaks within particular classrooms.

Another potential limitation is the presence of bias in reporting symptoms or high-risk contacts. In the case of young children, they may not recall whether they experienced symptoms during the study assessment period. However, efforts were made to mitigate this bias by collecting information from their legal guardians, predominantly parents. Additionally, it is possible that students and school staff were more aware of high-risk situations outside of school, primarily at home, which could explain the stronger association found when high-risk contacts occurred outside of school. Nevertheless, when considering the clustering effect (MOR) at the class and school level, this bias is less likely and the risk appears to be significantly smaller, confirming that schools provided a safer environment.

The observation that the schools may play a small role in SARS-CoV-2 transmission is relevant information to accurately ponder the pros and cons of school openings and to determine the most effective measures to mitigate transmission in schools and classrooms. However, the clustering effect found at the class level poses additional challenges to reduce the risk of infection among students, staff, and particularly to the community. For that, and although we cannot directly infer it from our study, measures such as improved ventilation, stricter hygiene protocols, vaccination promotion, and screening testing, among others^27^, might help to reduce within-class SARS-CoV-2 transmission.

In conclusion, there was a clustering effect within the classes but not within the school setting and the seroprevalence was much higher among individuals who had high-risk contacts outside school than among individuals who had high-risk contacts inside the school setting. Such findings indicate that it was possible to prevent the spread of outbreaks within classes to the rest of the school community.
